# Neoadjuvant systemic fluorouracil and mitomycin C prior to synchronous chemoradiation is an effective strategy in locally advanced rectal cancer

**DOI:** 10.1038/sj.bjc.6600822

**Published:** 2003-04-01

**Authors:** I Chau, M Allen, D Cunningham, D Tait, G Brown, M Hill, K Sumpter, A Rhodes, A Wotherspoon, A R Norman, A Hill, A Massey, Y Prior

**Affiliations:** 1 Department of Medicine, Royal Marsden Hospital, Downs Road, Sutton, Surrey SM2 5PT, UK; 2 Department of Radiotherapy, Royal Marsden Hospital, London Surrey, UK; 3 Department of Diagnostic Imaging, Royal Marsden Hospital, London Surrey, UK; 4 Department of Histopathology, Royal Marsden Hospital, London Surrey, UK; 5 Department of Computing, Royal Marsden Hospital, London Surrey, UK

**Keywords:** neoadjuvant chemotherapy, fluorouracil, radiotherapy, circumferential resection margin, magnetic resonance imaging

## Abstract

This study was designed to evaluate the benefits of neoadjuvant chemotherapy prior to chemoradiation and surgery in patients with locally advanced rectal cancer. Patients with previously untreated primary rectal cancer, reviewed in a multidisciplinary meeting and considered to have locally advanced disease on the basis of physical examination and imaging (MRI+CT *n*=30, CT alone *n*=6), were recruited. Patients received protracted venous infusion 5-FU (300 mg m^−2^ day^−1^ for 12 weeks) with mitomycin C (MMC) (7 mg m^−2^ i.v. bolus every 6 weeks). Starting on week 13, 5-FU was reduced to 200 mg m^−2^ day^−1^ and concomitant pelvic radiotherapy 45 Gy in 25 fractions was commenced followed by 5.4–9 Gy boost to tumour bed. Surgery was planned 6 weeks after chemoradiation. Postoperatively, patients received 12 weeks of MMC and 5-FU at the same preoperative doses. Between January 99 and August 01, 36 eligible patients were recruited. Median age was 63 years (range=40–85). Following neoadjuvant chemotherapy, radiological tumour response was 27.8% (one CR and nine PRs) and no patient had progressive disease. In addition, 65% of patients had a symptomatic response including improvement in diarrhoea/constipation (59%), reduced rectal bleeding (60%) and diminished pelvic pain/tenesmus (78%). Following chemoradiation, tumour regression occurred in 80.6% (six CRs and 23 PRs; 95% CI=64–91.8%) and only one patient still had an inoperable tumour. R0 resection was achieved in 28 patients (82%). When compared with initial clinical staging, the pathological downstaging rate in T and/or N stage was 73.5% and pathological CR was found in one patient. Neoadjuvant systemic chemotherapy as a prelude to synchronous chemoradiation can be administered with negligible risk of disease progression and produces considerable symptomatic response with associated tumour regression.

In patients with resectable carcinoma of the rectum, surgery remains the best option for cure. However, local recurrence rates of 25–40% have been reported in recent large series of patients undergoing conventional resection (Havenga *et al*, 1999; Nesbakken *et al*, 2002). Total mesorectal excision (TME), defined as a sharp dissection under clear vision with the excision of the rectum and mesorectum within the mesorectal fascia, has been adopted as the standard technique in rectal cancer by surgeons in several European countries although there is a lack of randomised data comparing TME with conventional surgery. Nevertheless, recurrence rates of <10% (Heald *et al*, 1998; Havenga *et al*, 1999; Nagtegaal *et al*, 2002; Wibe *et al*, 2002) and superior survival (Havenga *et al*, 1999; Kapiteijn *et al*, 2002) have been reported with TME. In rectal cancer surgery, circumferential resection margin (CRM) involvement, defined as tumour observed ⩽1 mm from the resection margin, has been shown to be an important prognostic factor resulting in both higher rates of local recurrence (Quirke *et al*, 1986; Adam *et al*, 1994; Birbeck *et al*, 2002; Nagtegaal *et al*, 2002; Wibe *et al*, 2002) and poor survival (Adam *et al*, 1994; Birbeck *et al*, 2002; Wibe *et al*, 2002) even after TME surgery (Nagtegaal *et al*, 2002).

Short-course preoperative radiotherapy (5 Gy daily for 5 days) has been shown to have a survival advantage and reduction in local recurrence compared to surgery alone in operable rectal cancer (Swedish Rectal Cancer Trial, 1997). Although only one trial has shown a survival advantage for preoperative radiotherapy (RT), its results have been found to be representative of that achieved in the general population (Dahlberg *et al*, 1999) leading to this approach being adopted by many oncologists in Europe. However, the value of preoperative RT in patients undergoing optimised TME surgery has been questioned and in a large randomised Dutch study of 1805 patients, preoperative RT has been shown to reduce local recurrence even when TME was used in all patients (Kapiteijn *et al*, 2001).

Preoperative chemoradiation (CRT) has the potential advantages of eliminating distant micrometastases at an early stage, enhancing radiosensitivity because of better oxygenated tissue, lowering incidence of acute toxicity compared with postoperative CRT and increasing sphincter preservation. The potential disadvantage of preoperative CRT is overtreatment of patients either because of early pathological stage (estimated to be 18% in one randomised study (Sauer *et al*, 2001)) or presence of occult metastatic disease un-detected on pretreatment imaging. Preoperative CRT has been used by many oncologists especially in North America for patients with clinical T3 disease based on extrapolated benefits from postoperative CRT and a number of nonrandomised studies demonstrating significant pathological complete response (pCR) rates and acceptable acute toxicity profile with the use of preoperative CRT. In patients with locally advanced, primarily irresectable cancer (i.e. a cancer where a complete gross surgical clearance is deemed unlikely to be achieved), preoperative CRT has been used to cause tumour regression to such an extent that the cancer can be removed radically with adequate clearance in the resection margin (Videtic *et al*, 1998; Chan *et al*, 2000; Rodel *et al*, 2000).

The principles on which our study was based were severalfold: neoadjuvant combination chemotherapy to (1) reduce the bulk of primary tumour, (2) delay the development of or eliminate micrometastases and (3) allow immediate commencement of anti-cancer treatment avoiding potential delay while waiting for definitive radiotherapy; preoperative synchronous chemoradiation to further reduce the bulk of the primary carcinoma leading to a higher R0 resection (i.e. resection with microscopic tumour clearance at resection margins) rate and a reduction of subsequent local recurrence; surgical resection of the primary tumour; and postoperative adjuvant chemotherapy to eliminate residual micrometa-stasis especially in those with R1 resection (microscopic incomplete resection with tumour present ⩽1 mm of the resection margin).

During neoadjuvant and adjuvant chemotherapy, a combination of mitomycin C (MMC) and protracted venous infusion (PVI) 5-fluorouracil (5-FU) was used in our study based on the *in vitro* synergy of these two drugs (Russello *et al*, 1989) and a superior response rate, failure-free survival and quality of life for this combination compared with PVI 5-FU alone in a previous randomised study of 200 patients with advanced colorectal cancer (Ross *et al*, 1997). Preoperative MMC, infused 5-FU/leucovorin and radiotherapy have also been shown to be an effective treatment for tethered/fixed rectal cancers (Chan *et al*, 2000). The objectives of this study were to evaluate the feasibility and benefits of delivering neoadjuvant chemotherapy prior to synchronous chemoradiation and surgery in patients with newly diagnosed locally advanced rectal cancer.

## PATIENTS AND METHODS

This study was approved by the local biomedical ethics committee. Signed, written informed consent was obtained from each patient.

### Patients selection and evaluation

The eligibility criteria were: locally advanced histologically proven adenocarcinoma of rectum; no previous chemotherapy or radiotherapy; no evidence of metastatic disease on clinical examination and radiological imaging; bidimensionally measurable disease; haemoglobin >10 g dl^−1^, white blood count >3 × 10^9^ l^−1^, neutrophil >1.5 × 10^9^ l^−1^, platelet >100 × 10^9^ l^−1^, bilirubin <30 *μ*mol l^−1^, creatinine <180 *μ*mol l^−1^ and calculated creatinine clearance >60 ml min^−1^.

Before entry into the study, all patients were assessed by our cancer network multi-disciplinary team comprising medical, radiation and surgical oncologists, gastroenterologists and radiologists. Patients were considered to have locally advanced disease on the basis of digital rectal examination and imaging (computed tomography (CT) or magnetic resonance imaging (MRI)). All patients had at least T3N0 disease on pretreatment clinical staging. All patients were required to have chest X-ray (CXR), CT scan of chest, abdomen and pelvis and carcinoembryonic antigen (CEA) measurement.

MRI scans of the pelvis were performed as previously described (Brown *et al*, 1999) in patients who could tolerate the procedure and had no contraindications to MRI, but were not mandated in the protocol because of inaccessibility to urgent staging MRI from some referring clinicians at the beginning of the study. However, MRI scans were obtained in the majority of enrolled patients. MRI criteria for locally advanced disease were: tumour extending to within 1 mm of or beyond the mesorectal fascia (i.e. CRM involved or threatened); T3 low-lying tumour at or below the levators, tumour extending 5 mm or more into perirectal fat, T4 tumours and T1-4N2 tumours. Information from both imaging and digital rectal examination was considered complimentary to give final staging.

### Treatment

Figure 1

**Figure 1:**
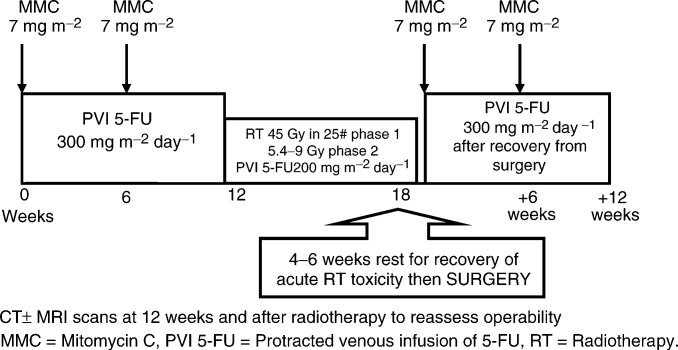
Treatment schema.

shows the overall treatment schema.

#### Neoadjuvant chemotherapy

Twelve weeks of neoadjuvant chemo-therapy was given. Mitomycin C (7 mg m^−2^) was delivered as a bolus injection repeated every 6 weeks, thus, a total of two doses were given during this period. Screening of the peripheral blood film for red cell fragmentation, indicating a risk of developing haemolytic uraemic syndrome with further MMC therapy, was mandated before each course of MMC. A maximum dose of 14 mg of MMC was allowed in each course. 5-FU (300 mg m^−2^ day^−1^) was administered as a continuous infusion via a central venous catheter (Hickman line). No routine antiemetic medications were given. Warfarin (1 mg day^−1^ orally) was administered throughout the treatment to prevent catheter thrombosis.

#### Dose modifications

Toxicity was assessed according to National Cancer Institute–Common Toxicity Criteria (NCI–CTC) version 2 (1998). Toxicity data were collected weekly during chemotherapy. If grade 3 and 4 neutropenia occurred, subsequent doses of MMC were reduced by 25 and 50%, respectively. If stomatitis, hand–foot syndrome or diarrhoea relating to 5-FU developed, 50, 100 and 150 mg m^−2^ dose reductions in 5-FU were made if grade 2, 3 and 4 toxicities developed, respectively.

#### Synchronous chemoradiation (CRT)

On completion of 12 weeks' neoadjuvant chemotherapy, patients began chemoradiation. This was delivered by a two-phase technique, both phases were CT planned and involved the use of customised blocking on all fields. Phase 1 delivered a total of 45 Gy in 25 daily fractions, each of 1.8 Gy over 5 weeks and encompassed the primary tumour and pelvic lymph nodes. The superior margin was at the level of L5/S1 while the inferior margin varied depending on the position of the tumour within the rectum, but with a minimum of 3 cm margin on the inferior extent. Laterally, the pelvic side walls, plus 1 cm, were covered and the sacrum was included posteriorly. The anterior margin depended on the position and extent of the tumour. During phase 2, the protocol aim was to deliver 9 Gy in five fractions covering the tumour either clinically palpable or visible on imaging with a 2 cm margin in all directions. Where CT planning indicated that small bowel could not be adequately excluded from this volume, the dose was modified to 5.4 Gy in three fractions. The information used to define the phase 2 target was the pretreatment CT scan, pretreatment clinical evaluation and, where available, pretreatment MRI.

Both the phase 1 and 2 were delivered by three field techniques, a posterior and two laterals or two lateral obliques. Patients were treated prone with a full bladder and received concomitant PVI 5-FU at a reduced dose of 200 mg m^−2^ day^−1^ throughout radiotherapy. If patients already had dose reduction of 5-FU to below 200 mg m^−2^ day^−1^ during neoadjuvant chemotherapy, that same reduced dose of 5-FU would be applied during synchronous chemoradiation.

#### Dose modifications

Acute toxicity was assessed according to Radiation Therapy Oncology Group–Acute Radiation Morbidity Scoring Criteria. Toxicity data were collected weekly during radiotherapy and then 1 month after radiotherapy. If toxicity because of 5-FU occurred during CRT, the dose was adjusted as outlined in the neoadjuvant chemotherapy section.

#### Surgery

Surgery was performed 6 weeks after the completion of CRT. The choice of surgical procedure (abdomino-perineal resection or anterior resection) was at the surgeons' discretion.

#### Postoperative adjuvant chemotherapy

An identical 12 week block of postoperative chemotherapy, consisting of MMC and PVI 5-FU at the same preoperative doses, was given to all patients who had recovered within 12 weeks of surgery and had no evidence of distant disease postoperatively.

### Evaluation of response

Clinical tumour response was measured using CT and MRI scans. CT scans were repeated after the initial neoadjuvant chemotherapy at 12 weeks, after synchronous chemoradiation at 22 weeks (i.e. 4 weeks after finishing RT) and before commencement of postoperative adjuvant chemotherapy. The primary intention of CT scan was to exclude any development of distant metastasis. MRI scans of pelvis were repeated once after synchronous chemoradiation to assess primary tumour response. All available imaging was reviewed independently by one radiologist (GB), who was blinded to the pathological findings. The local T and N stage and tumour measurement were made according to previously published criteria (Brown *et al*, 1999). No confirmatory scans for responses were performed.

Radiological tumour response was evaluated according to World Health Organisation (WHO) Criteria (Miller *et al*, 1981). Complete response (CR) was defined as the complete disappearance of all measurable lesions, without the appearance of new lesion(s). Partial response (PR) was defined as a reduction of bidimensional lesions by ⩾50% of the sum of the products of the largest perpendicular diameters of each measurable lesion and no progression in other lesions or the appearance of any new lesions. Stable disease (SD) was defined as a <50% reduction of tumour volume or a <25% increase of the volume of one or more measurable lesions, with no new lesions. Progressive disease (PD) was defined as an increase of ⩾25% of the size of at least one bidimensionally measurable lesion, the appearance of new lesion(s), and/or the onset of ascites or pleural effusion with cytological confirmation.

During neoadjuvant chemotherapy, tumour-related symptoms were assessed by research nurses with a 15-point checklist at baseline and at each hospital visit for patients who had these symptoms on entry into study. Particular enquiry was made regarding symptoms of rectal bleeding, pelvic pain/tenesmus and diarrhoea/constipation. Disappearance or attenuation of these tumour-related symptoms were recorded at each hospital visit. Data regarding the time between commencement of treatment and resolution of symptoms were collected weekly. This symptom checklist has been used in a number of previous multicentre randomised studies (Ross *et al*, 1997,^2002^; Cunningham *et al*, 1998; Maisey *et al*, 2002; Tebbutt *et al*, 2002).

Pathological response was assessed by examining the resected tumour specimen after chemoradiation and compared with baseline clinical staging using imaging and digital rectal examination. The American Joint Committee on Cancer TNM staging system (fifth edition) was used when assessing for pathological response. Tumour downstaging was defined as a reduction of at least one level in T or N staging (e.g. T3 to T2, N2 to N0). Tumour specimens were also examined for resection margin involvement. CRM involvement was defined as tumour observed ⩽1 mm from the resection margin.

### Follow-up

Patients were seen in the routine follow-up clinic every 3 months for the first year, every 6 months for the second year and then annually. CEA measurement was performed with each clinic visit. CT scans of thorax, abdomen and pelvis were performed 1 year and 2 years after the end of treatment.

### Statistical analysis

Failure-free survival and overall survival were estimated using the Kaplan–Meier method from trial entry (Kaplan and Meier, 1958). All end points were updated in May, 2002. Failure-free survival was calculated from the date chemotherapy commenced to the date of either disease progression or death. Overall survival was estimated from the date chemotherapy commenced to the date of death from any cause.

## RESULTS

In all, 36 eligible patients were recruited between January 1999 and August 2001. The median follow-up for these patients is 15 months. Table 1

**Table 1:** Patient characteristics

**Patient characteristics**	**Number of patients (total *n*=36)**
*Gender*	
Male	26
Female	10
	
Median age (range)	63 years (40–85)
	
*Performance status*	
0	13
1	23

shows the patient demographics. At baseline, both MRI and CT scan were carried out in 30 patients and CT scans alone were performed in six patients. Table 2

**Table 2:** Baseline staging using CT±MRI scans and digital rectal examination

**Baseline staging**	**Number of patients (%) *n*=36**	**Number of patients with CRM involved or threatened by tumour *n*=11**
T2N0^a^	1 (2.8)	0
T3N0	6 (16.7)	1
T3N1	8 (22.2)	2
T3N2	6 (16.7)	6
T4N0	8 (22.2)	2
T4N1	5 (13.9)	0
T4N2	2 (5.6)	0

CRM=circumferential resection margin.

a T3N1 on initial MRI reporting at trial entry, but subsequently reclassified after radiology review.

shows the baseline clinical staging. One patient had T3N1 rectal cancer on initial MRI report and was enrolled into the trial, but was subsequently reclassified as T2N0 after radiology review. This patient was included in all analyses. Eleven patients had the potential mesorectal CRM threatened or involved by tumour on MRI at baseline. Of those, CRM was threatened by nodal or extranodal tumour deposits rather than by primary tumour directly in five patients.

Figure 2

**Figure 2:**
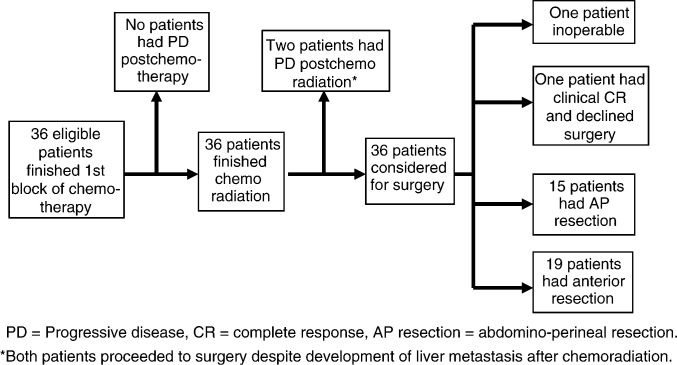
Progress of all patients during trial.

shows the progress of all patients during the trial. No patient developed detectable progressive disease after neoadjuvant chemotherapy. In two patients, liver metastases were evident on CT after synchronous chemoradiation. No progression in primary tumours was seen during the trial.

### Tumour response

#### Radiological response

All 36 patients were evaluable for radiological response (Table 3

**Table 3:** Objective tumour responses by imaging

	**Post chemotherapy (CT only)**	**Post chemoradiation (CT±MRI)**
Complete response	1 (2.8%)	6 (16.7%)
Partial response	9 (25%)	23 (63.9%)
Stable disease	26 (72.2%)	5 (13.9%)
Progressive disease	0 (0%)	2 (5.6%)
Objective response rates (95% confidence interval)	27.8% (14.2–45.2%)	80.6% (64–91.8%)

). CT scans were performed on all 36 patients after neoadjuvant chemotherapy and synchronous CRT. Pelvic MRI scans were performed in 32 patients only after CRT. Four patients did not have post-CRT MRI scans because of patient refusal (*n*=2), *in situ* coronary stent (*n*=1) and unavailability of MRI (*n*=1).

After neoadjuvant chemotherapy, the best achieved objective response rate (ORR) of all patients were 27.8% (95% confidence interval [CI]: 14.2–45.2%) with one CR and nine PRs. After chemoradiation, the objective response rate was 80.6% (95% CI: 64–91.8%) with six CRs and 23 PRs.

#### Resolution of symptoms

Overall 65% of patients had an improvement or resolution of symptoms. Of the patients with symptoms, 59% had improvement in diarrhoea/constipation, 60% had reduced rectal bleeding and 78% had diminished pelvic pain and tenesmus. All symptomatic improvement was evident during neoadjuvant chemotherapy. The median time to improvement in diarrhoea/constipation was 28 days (interquartile range=7–43 days) and for diminished pelvic pain and tenesmus was 35 days (interquartile range=7.5–56 days).

#### Surgery and pathological response

Nineteen patients underwent an anterior resection and 15 had an abdomino-perineal resection. Patients proceeded to surgery in a median of 6.9 weeks after finishing RT. One patient with T4N1 tumour was found to be still inoperable at laparotomy and no attempt of surgical resection was made. One 85-year-old patient achieved a clinical complete response on both imaging and sigmoidoscopic evaluation and declined surgery after CRT. He developed local recurrence 14 months later and underwent a successful TME with complete tumour clearance. Both patients who developed liver metastases after CRT opted to undergo resection of primary tumour before receiving further palliative chemotherapy. One patient was found to have metastases on the serosal surface of liver at operation undetected on preoperative CT. Thus, potentially curative surgery was attempted on 33 patients. Another patient was found to have a rise in CEA level during the postoperative recovery period. A positron emission tomography (PET) scan demonstrated widespread metastatic disease without evidence of active disease on postoperative CT.

Compared with baseline staging, 25 patients (73.5%) had downstaging of their primary tumour on histological examination either in T (*n*=13) or N (*n*=7) or both (*n*=5) staging. Pathological CR was found in one patient. Table 4

**Table 4:** Pathological response

	**Pathological staging**				
**Baseline staging**	**pT0**	**pT1**	**pT2**	**pT3**	**pT4**
T2	0	0	1	0	0
T3	0	3	4	11	1
T4	1	0	2	8	3
					
		pNode negative		pNode positive	
Node negative	12	2			
Node positive	8	12			

shows the pathological response in patients who underwent resection of their primary tumour. The median number of lymph nodes retrieved in the surgical specimens was 5 (range 0–17). In one patient only, no lymph nodes were identified (Nx) from the surgical specimen after CRT.

R0 resections were performed in 28 out of 34 patients (82%). Of the 11 patients with threatened or involved CRM on baseline MRI scan, nine had tumour regression from the resection margin after CRT. In only one patient, tumour was predicted to have regressed from CRM on postCRT MRI, but histology showed involved resection margin.

### Postoperative adjuvant chemotherapy

Twenty-two patients (61%) received adjuvant chemotherapy. Fourteen patients did not receive adjuvant chemotherapy because of postoperative complications (*n*=5), progressive disease or inoperable tumour (*n*=5) and physicians' or patients' decision (*n*=4). One patient developed venous thrombosis secondary to Hickman line and received capecitabine instead of PVI 5-FU during adjuvant chemotherapy.

### Toxicity

#### Neoadjuvant chemotherapy-induced toxicity

Table 5

**Table 5:** Treatment induced grade 3/4 toxicity

**Toxicity**	**Number of patients**
*During neoadjuvant chemotherapy*	
Anaemia	0 (0%)
Neutropenia	0 (0%)
Thrombocytopenia	0 (0%)
Diarrhoea	3 (8.3%)
Stomatitis	1 (2.8%)
Hand–foot syndrome	4 (11%)
Infection	2 (5.6%)
Febrile neutropenia	0 (0%)
	
*During chemoradiation*	
Anaemia	0 (0%)
Neutropenia	0 (0%)
Thrombocytopenia	0 (0%)
Lower gastrointestinal	0 (0%)
Genitourinary	0 (0%)
Skin	10 (27.8%)

shows the incidences of grade 3/4 toxicities during neoadjuvant chemotherapy. There were no deaths related to chemotherapy. No grade 3/4 haematological toxicity was seen. Nine patients (25%) developed grade 3/4 nonhaematological toxicity although only 3% were grade 4. The most common nonhaematological toxicity was hand–foot syndrome.

#### Chemoradiation-induced toxicity

Table 5 shows the incidences of grade 3/4 toxicities during chemoradiation. There were no deaths related to chemoradiation either. The most frequent toxic effect was treatment field erythema. In most cases, this had resolved when patients were reviewed one month after completion of radiotherapy. No haematological, lower gastrointestinal and genitourinary grade 3/4 toxicities were encountered during chemoradiation. No treatment interruption was required.

#### Surgical complications

One patient died postoperatively from an anastamotic leak leading to multiorgan failure. No other anastamotic leak was seen. Five other patients developed postoperative complications including pelvic collections (*n*=3) and delayed wound healing (*n*=2).

### Survival

**Figure 3:**
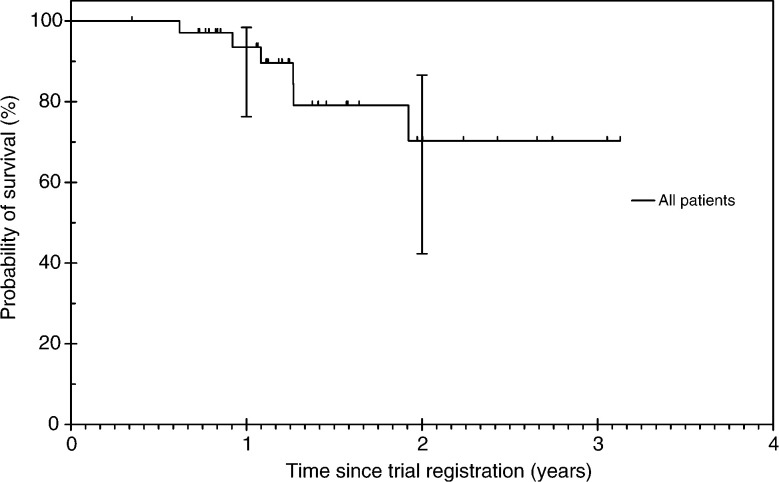
Overall survival.

Six out of 36 patients (16.7%) have died. Cause of death was progressive cancer in all cases. The median survival has not yet been reached (Figure 3

). The survival probability at 1 year was 93.5% (95% CI: 76.3–98.4%) and at 2 years was 70.3% (95% CI: 42.3–86.6%).

### Patterns of failure

**Figure 4:**
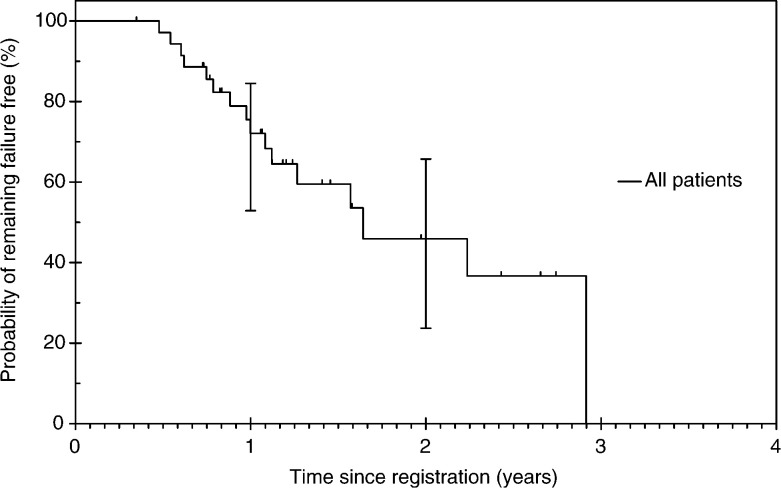
Failure-free survival.

The median failure-free survival was 18.6 months (Figure 4

). The failure-free survival probability at 1 year was 72.1% (95% CI: 52.9–84.5%). Two patients had local recurrences, nine developed distant metastasis (lung *n*=3, liver *n*=4, brain *n*=1 and paraaortic lymphadenopathy *n*=1) and one had both local and distant disease as their first sites of treatment failure.

## DISCUSSION

In this study, we assessed the feasibility of delivering neoadjuvant chemotherapy before preoperative CRT and radical resection. This treatment strategy potentially addresses systemic micrometastases as well as reduces the frequency of locoregional recurrence. A reduction in the size of the primary tumour with neoadjuvant chemotherapy may have improved the effectiveness of chemo-radiotherapy and also increased R0 resection rate. This is supported by the fact that 41% of patients included in our study had T4 tumours and a further 33% had tumour extending to the potential mesorectal CRM in whom resection with curative intent would not normally be attempted. Of these patients, 77% underwent a R0 resection. Moreover, neoadjuvant chemotherapy resulted in rapid symptom resolution which would impact on patients' quality of life. Antitumour treatment could also be started in a timely fashion without potential delay as long course radical radiotherapy could take two months to commence in the United Kingdom.

Several strategies have been used to reduce either local recurrence or distant metastasis for localised rectal cancer. Improved surgical technique such as total mesorectal excision has been reported to have a lower local recurrence rate and improved survival compared to conventional surgery (Havenga *et al*, 1999; Kapiteijn *et al*, 2002). The value of preoperative RT in operable rectal cancer has been evaluated in two meta-analyses (Camma *et al*, 2000; Colorectal Cancer Collaborative Group, 2001). Whereas the Colorectal Cancer Collaborative Group, using individual data from 6350 patients enrolled in 13 randomised studies, found a marginal but nonsignificant survival advantage in patients receiving preoperative RT (Colorectal Cancer Collaborative Group, 2001), a significant reduction in mortality was found by the meta-analysis undertaken by Camma *et al* (2000). Both meta-analyses demonstrated a significant reduction in local recurrence with preoperative RT. The role of preoperative RT was further examined in the Dutch TME trial in which the surgical technique was standardised (Kapiteijn *et al*, 2001). Although no significant difference in 5-year survival was seen between the two arms, the local recurrence rate in the preoperative RT group (5.8%) was significantly lower than in the surgery alone group (11.6%) (Van de Velde, 2002).

At least three randomised studies of preoperative *vs* postoperative chemoradiation have been conducted, but the two studies from the US (INT-0147 and NSABP R-03) closed prematurely because of poor accrual. In the National Surgical Adjuvant Breast and Bowel Project (NSABP) R-03 trial with only 267 patients randomised, a larger proportion of patients receiving preoperative treatment had sphincter saving surgery (44 *vs* 34%) and had no evidence of disease at 1 year compared to those receiving postoperative treatment (Roh *et al*, 2001). However, increased toxicity and a slight increase in early deaths were seen in the preoperative arm. The German CAO/ARO/AIO-94 study showed that preoperative chemoradiation was well tolerated and carried no higher risk of postoperative morbidity, but efficacy data are awaited (Sauer *et al*, 2001).

Although many studies evaluated the use of preoperative chemoradiation in patients with newly diagnosed locally advanced rectal cancer, the definition of locally advanced disease varies between studies (Glimelius, 2001). Many recent studies included T3 tumours staged by endoscopic ultrasound which are often less bulky than clinically staged T3 tumours. Very few patients with T4 tumours were recruited (<5% of total enrolled) in these studies (Janjan *et al*, 1999; Bosset *et al*, 2000; Chan *et al*, 2000; Grann *et al*, 2001; Onaitis *et al*, 2001; Valentini *et al*, 2001). In our study, over 40% of patients had T4 tumours representing a group of patients with truly locally advanced disease. This may account for the low pathological complete response rate seen in our study compared with 10–30% achieved in other studies (Janjan *et al*, 1999; Bosset *et al*, 2000; Chan *et al*, 2000; Grann *et al*, 2001; Onaitis *et al*, 2001; Valentini *et al*, 2001). Two studies included only patients with clinically staged T4 tumours (Videtic *et al*, 1998; Rodel *et al*, 2000). The pathological CR rate was lower (Rodel *et al*, 2000) and R0 resection was less frequently achieved (Videtic *et al*, 1998) compared to other published studies despite the use of higher dose radiotherapy. Toxicity was significant in one study with 16% not completing protocol (Rodel *et al*, 2000). Patients with T or N downstaging have been shown to have a significantly improved local control, freedom from distant metastasis, disease-free survival and overall survival (Valentini *et al*, 2002). Despite a low pCR rate, our pathological downstaging rate of 74% would be clinically meaningful to this group of patients with advanced disease.

In our study, the radiological tumour response rate was 28% after neoadjuvant chemotherapy increasing to 81% after chemo-radiation. Although the radiation component would have contributed to the greatly improved response rate after chemoradiation, it is conceivable that CT imaging, that was used primarily to exclude distant spread after neoadjuvant chemo-therapy, might have underestimated the primary tumour response compared to MRI that was used after chemoradiation. A clinical response after preoperative CRT in rectal cancer has been shown to predict significantly better long-term clinical outcomes (Valentini *et al*, 2002). This supports the use of preoperative tumour assessment by imaging rather than simply relying on pathological downstaging as an efficacy outcome measure. However, the accuracy of MRI in the assessment of the primary rectal cancer after chemotherapy or chemoradiation has not been examined extensively. Continuing evaluation of MRI, positron emission tomography and endoscopic ultrasound after neoadjuvant treatment as a guide to surgical management may allow more conservative approach for responding patients. However as noted in our study and other studies (Hiotis *et al*, 2002), many patients with clinical CR had persistent foci of tumours that were not detectable on preoperative imaging, therefore treatment decisions should not be based solely on the absence of clinically palpable or visible tumour after chemoradiation. Indeed, one patient with clinical CR in our study was found to have residual tumour and CRM involvement following resection highlighting the risk of no excision after obtaining a clinical CR.

The ability of MRI to accurately stage rectal cancer (Brown *et al*, 1999; Beets-Tan *et al*, 2001), define the potential mesorectal circumferential margin (Brown *et al*, 1999) and predict CRM involvement (Beets-Tan *et al*, 2001) has been demonstrated before and supported its use in the initial staging of patients in our study. Encouragingly, over 80% of patients in our study with CRM threatened or involved initially demonstrated tumour regression from the CRM after treatment, thus allowing a greater proportion of curative surgery to be performed. Reassuringly, the prediction of CRM involvement after treatment by MRI was relatively accurate in our study and might be used to guide surgical management after neoadjuvant treatment in the future. Other MRI features such as tumour thickness, tumour appearances, extramural spread may give complimentary information about tumour response in addition to TNM staging. An analysis of MRI features at baseline and postchemoradiation and their correlation with pathological findings and survival for patients undergoing a similar treatment programme in our institution has been performed and will be reported separately. An ongoing study in Europe (MERCURY, Magnetic Resonance Imaging and Rectal Cancer European Equivalence Study) is designed to correlate MRI findings on extramural spread and potential CRM involvement with pathological specimen and will provide valuable information on the use of MRI for rectal cancer.

Direct comparison of our efficacy results with other studies would be problematic because of differences in the patient population (e.g. proportion of patients with T4 or node-positive tumours), and in the doses and schedules of drugs used in the preoperative CRT. The follow-up in our study is also relatively short to assess the impact of our treatment programme on survival. Rather, we have demonstrated the feasibility of using neoadjuvant chemotherapy prior to synchronous CRT, and the immediate benefits associated with its use such as tumour response, resolution of symptoms, low risk of disease progression and R0 resectability in many patients.

Neoadjuvant chemotherapy with MM C and PVI 5-FU was well tolerated with no unexpected toxicity, the incidence of side effects was similar to that reported in randomised studies (Ross *et al*, 1997; Maisey *et al*, 2002; Tebbutt *et al*, 2002) and it also did not increase the frequency of severe adverse events during CRT. The low incidence of grade 3/4 lower gastrointestinal and genitourinary toxicity during CRT might be related to the fact that patients underwent 12 weeks of neoadjuvant chemotherapy prior to CRT, therefore patients who were susceptible to fluorouracil-related toxicity would have had appropriate dose reductions already.

However, our strategy will require further refinement. A more effective approach would be incorporation of newer chemotherapy agents as distant metastasis is the most frequent cause of our treatment failures. Oxaliplatin and infused 5-FU/leucovorin (LV) has shown considerable antitumour activity in randomised phase III studies (de Gramont *et al*, 2000; Giacchetti *et al*, 2000) and has recently been found to be superior to irinotecan/bolus 5-FU/LV in terms of efficacy and toxicity profile in metastatic colorectal cancer (Goldberg *et al*, 2002). Concomitant oxaliplatin, infused 5-FU/ LV and radiotherapy have been reported in locally advanced rectal cancer with a pathological complete response rates of 14–29% (Freyer *et al*, 2001; Aschele *et al*, 2002; Gerard *et al*, 2002; Sebag-Montefiore *et al*, 2002). Capecitabine has also been combined with oxaliplatin (Borner *et al*, 2002; Taberno *et al*, 2002) or radiotherapy (Dunst *et al*, 2002; Kim *et al*, 2002) and yielded promising activity in colorectal cancer. In our current active protocol, we have elected to substitute MMC and PVI 5-FU with oxaliplatin and capecitabine during the neoadjuvant chemotherapy and use capecitabine as the radiosensitising agent during chemoradiation.

In conclusion, neoadjuvant systemic chemotherapy prior to synchronous chemoradiation can be administered with negligible risk of local disease progression and low risk of systemic spread. It produced considerable symptomatic response with associated tumour regression. This treatment programme allowed sufficient tumour shrinkage for R0 resection in the majority of our patients with locally advanced rectal cancer including those with initial circumferential resection margin involvement.
